# Supplementary material for: Content-rich biological network constructed by mining PubMed abstracts
Source: BMC Bioinformatics. 2004 Oct 8;5:147. doi: 10.1186/1471-2105-5-147 (PMC528731; doi:10.1186/1471-2105-5-147)
Supplement: Additional File 2 — The original results of the above study (non-essential files are deleted to keep the file size under the limit set by BMC bioinformatics). [file 1471-2105-5-147-S2.bz2 › chilibotAdditionalFile2/dip20/578ID9204896E10946/html/TEK_ANGPT1.html]

 


 **TEK** and **ANGPT1** 
  
Found 178 abstracts in PubMed, retrieved 20.  
 

 What does Google say? 
 PDF only 
| .edu only 

---

**Interactive relationship** (e.g. stimulation, inhibition, etc)

**Neutral relationship**- Recent studies have implicated the Tie2  [ **TEK** ]  tyrosine kinase receptor and its main ligands angiopoietin 1  [ **ANGPT1** ]  Ang 1  [ **ANGPT1** ]  and angiopoietin 2 Ang  [ **ANGPT1** ]  2 as crucial regulators of mural cell recruitment during angiogenesis.  Ref: 12876214 J Cell SciJ Cell Sci,
- Inhibition of interaction of VEGFR 2 or VEGFR 3 with VEGF D but not of Tie 2  [ **TEK** ]  angiopoietin 1  [ **ANGPT1** ]  interaction with soluble receptors abrogated Akt activation and survival of TEC.  Ref: 12709414 FASEB J< MedlineTA>FASEB J,
- These data provide comprehensive evidence that VEGFR 1 and 2, and angiopoietin 1  [ **ANGPT1** ] , angiopoietin 2 and Tie 2  [ **TEK** ]  interactions may be involved in the preparation of endometrium for implantation, remodelling of the maternal vasculature and trophoblast invasion during the peri implantation period in this primate species.  Ref: 12887279 Reproduction, 2003
- Two of the Angs, Ang 1  [ **ANGPT1** ]  and Ang  [ **ANGPT1** ]  4, activate the Tie2  [ **TEK** ]  receptor.  Ref: 12783598 Expert Opin Investig Drugs, 2003
- Angiopoietins Ang  [ **ANGPT1** ]  have been recently identified as ligands for Tie 2  [ **TEK** ]  receptor and are thought to be important factors in vascular maturation and stability during angiogenesis.  Ref: 12717391 Hepatology, 2003
- 1 Angiopoietin 1  [ **ANGPT1** ]  Ang1  [ **ANGPT1** ]  is an angiogenic growth factor that binds to the Tie2  [ **TEK** ]  receptor on vascular endothelium, promoting blood vessel maturation and integrity.  Ref: 12770938 Br J Pharmacol, 2003
- Tie 2  [ **TEK** ]  dependent activation of RhoA and Rac1 participates in endothelial cell motility triggered by angiopoietin 1  [ **ANGPT1** ] .  Ref: 12816861 Blood< MedlineTA>Blood, 2003
- The angiopoietin Ang  [ **ANGPT1** ]  family of growth factors includes four members, all of which bind to the endothelial receptor tyrosine kinase Tie2  [ **TEK** ] .  Ref: 12783598 Expert Opin Investig Drugs, 2003
- ... Ang  [ **ANGPT1** ]  2 and Ang  [ **ANGPT1** ]  3 inhibit Ang 1  [ **ANGPT1** ]  induced Tie2  [ **TEK** ]  phosphorylation.  Ref: 12783598 Expert Opin Investig Drugs, 2003
- Ang 1  [ **ANGPT1** ]  activates the endothelial cell specific tyrosine kinase receptor Tie 2  [ **TEK** ] , which in turn leads to enhanced endothelial cell survival and stabilization.  Ref: 12810673 Cancer Res, 2003
- Using a combination of immunohistochemistry, laser capture microdissection and real time quantitative reverse transcription PCR, we showed that tumor derived VEGF significantly up regulated the expression of Ang  [ **ANGPT1** ]  2 in host stroma endothelial cells, resulting in markedly increased Ang  [ **ANGPT1** ]  2 Tie 2  [ **TEK** ]  mRNA copy number ratio in vivo.  Ref: 12810677 Cancer Res, 2003

**Non-interactive relationship** (e.g. studied together, co-existance, homology, etc.)

- To investigate the specific role of Ang2 in the adult vasculature, we generated a nuclease resistant RNA aptamer that binds and inhibits Ang2 but not the related Tie2  [ **TEK** ]  agonist, angiopoietin 1  [ **ANGPT1** ] .  Ref: 12692304 Proc Natl Acad Sci U S A, 2003
- In conclusion, Ang2 and Tie2  [ **TEK** ]  expressions increased after ischemia reperfusion in the rat ventricular myocardium, while Ang1  [ **ANGPT1** ]  and Tie1 expressions did not.  Ref: 12737621 Clin Sci Lond < MedlineTA>Clin Sci Lond , 2003
- The purpose of this study was to evaluate the expression of Tie 2  [ **TEK** ]  and Ang 1  [ **ANGPT1** ]  and 2 in an acetic acid induced gastric ulcer healing process in rats.  Ref: 12768384 J Gastroenterol, 2003
- mRNAs for VEGF, VEGFR 1 and VEGFR 2, angiopoietin 1  [ **ANGPT1** ] , angiopoietin 2 and their receptor Tie 2  [ **TEK** ]  were localized and quantified by in situ hybridization.  Ref: 12887279 Reproduction, 2003
- In conclusion, our data suggest that both hypoxia and VEGF treatment differentially regulate the angiopoietin Tie2  [ **TEK** ]  system in the two vascular cells and that, particularly in BRP, the regulation of Ang1  [ **ANGPT1** ] , but not Ang2, and Tie2  [ **TEK** ]  expression may play an important role in vascular development.  Ref: 12686170 Microvasc Res, 2003
- METHODS Since a link has been established in neoplasias between tumor growth and an increased expression of angiogenic growth factors, 136 samples of chorioangiomas and 136 samples of tumor free placental tissue were examined in terms of proliferation rate and expression of the growth factors angiopoietin 1  [ **ANGPT1** ]  and 2, the angiopoietin receptor Tie 2  [ **TEK** ] , PDGF and the PDFG beta receptor.  Ref: 12747234 J Perinat Med, 2003
- Tie 2  [ **TEK** ]  receptor expression was markedly downregulated in rats treated with MCT, and this was partially restored by gene therapy with Ang 1  [ **ANGPT1** ] .  Ref: 12690034 Circ Res, 2003
- Angiopoietin 1  [ **ANGPT1** ]  Ang 1  [ **ANGPT1** ] , a newly discovered ligand of the endothelial specific tyrosine kinase receptor Tie 2  [ **TEK** ] , has been found to promote cell survival, vascular maturation, and stabilization.  Ref: 12690034 Circ Res, 2003
- However, using bovine aortic endothelial cells BAEC, we found that only Ang2 expression, but neither Ang1  [ **ANGPT1** ]  nor Tie2  [ **TEK** ] , responded to these two angiogenic stimuli, which was consistent with many previous reports.  Ref: 12686170 Microvasc Res, 2003
- Using RT PCR and immunohistochemistry we demonstrate that testicular vascular endothelial growth factor A VEGF A, ang 1  [ **ANGPT1** ] , ang  [ **ANGPT1** ]  2 and the tie 2  [ **TEK** ]  angiopoietin receptor are expressed in the testis.  Ref: 12773423 Biol Reprod< MedlineTA>Biol Reprod, 2003
- The Tie 2  [ **TEK** ]  receptor tyrosine kinases and ligands, angiopoietin Ang 1  [ **ANGPT1** ]  and 2 have been postulated to play key roles in vascular development.  Ref: 12768384 J Gastroenterol, 2003
- In this study, we investigated the expression of Ang 1  [ **ANGPT1** ] , Ang  [ **ANGPT1** ]  2, Tie 2  [ **TEK** ] , and vascular endothelial growth factor VEGF in surgically resected specimens from 46 patients with HCC to determine their potential role in tumor angiogenesis and its progression.  Ref: 12717391 Hepatology, 2003
- Immunoprecipitation showed phosphorylation of MPC Tie2  [ **TEK** ]  on tyrosine residues upon stimulation with Ang 1  [ **ANGPT1** ]  or Ang  [ **ANGPT1** ]  2.  Ref: 12876214 J Cell SciJ Cell Sci, 2003
- However, the underlying mechanism for Ang1  [ **ANGPT1** ]  Tie2  [ **TEK** ]  signaling is not fully understood.  Ref: 12890486 Biochem Biophys Res Commun, 2003
- Localization of Tie2  [ **TEK** ]  and phospholipase D in endothelial caveolae is involved in angiopoietin 1  [ **ANGPT1** ]  induced MEK ERK phosphorylation and migration in endothelial cells.  Ref: 12890486 Biochem Biophys Res Commun, 2003
- 2 PLD is a new mediator of Ang1  [ **ANGPT1** ]  Tie2  [ **TEK** ]  induced signaling pathway, and it participates in MAPK activation and endothelial cell migration.  Ref: 12890486 Biochem Biophys Res Commun, 2003
- 0.01 but not between VEGF and Ang 1  [ **ANGPT1** ]  or Tie 2  [ **TEK** ]  in human ovarian cancer specimens.  Ref: 12810677 Cancer Res, 2003
- These findings suggest that Tie 2  [ **TEK** ]  and Ang 1  [ **ANGPT1** ]  and 2 play an important role in angiogenesis in the early phase of ulcer healing.  Ref: 12768384 J Gastroenterol, 2003
- Expression of Tie 2  [ **TEK** ]  and Ang 1  [ **ANGPT1** ]  and 2 mRNAs was detected in normal gastric tissue and ulcerative tissues by RT PCR.  Ref: 12768384 J Gastroenterol, 2003
- Hypoxia and vascular endothelial growth factor acutely up regulate angiopoietin 1  [ **ANGPT1** ]  and Tie2  [ **TEK** ]  mRNA in bovine retinal pericytes.  Ref: 12686170 Microvasc Res, 2003
- The study of angiopoietin 1  [ **ANGPT1** ]  Ang 1  [ **ANGPT1** ]  and angiopoietin 2 Ang  [ **ANGPT1** ]  2, ligands of receptor tyrosine kinase 2 Tie 2  [ **TEK** ] , showed that while stroma derived Ang  [ **ANGPT1** ]  2 was increased, epidermal Ang 1  [ **ANGPT1** ]  expression was completely abolished at early papilloma formation.  Ref: 12766907 Mol Carcinog, 2003
- On the other hand, Ang 1  [ **ANGPT1** ]  and Tie 2  [ **TEK** ]  mRNA expression in HCC was not different from that in adjacent liver tissue.  Ref: 12717391 Hepatology, 2003
- Expression of Tie 2  [ **TEK** ]  and angiopoietin 1  [ **ANGPT1** ]  and 2 in early phase of ulcer healing.  Ref: 12768384 J Gastroenterol, 2003
- Angiopoietin 1  [ **ANGPT1** ]  Ang1  [ **ANGPT1** ]  and its receptor, Tie2  [ **TEK** ] , play critical roles in blood vessel formation.  Ref: 12890486 Biochem Biophys Res Commun, 2003
- Polyclonal antibodies specific for Ang1  [ **ANGPT1** ] , Ang2, Tie2  [ **TEK** ]  and VEGF were used for immunostaining.  Ref: 12815197 Ophthalmic Res, 2003
- Angiopoetin 1 Ang1  [ **ANGPT1** ] , angiopoietin 2 Ang2, Tie 1, and Tie 2  [ **TEK** ]  were measured immediately after relief of occlusion, 60 min, 4, and 72 h after reperfusion by Northern blot, Western blot, and immunohistochemical staining.  Ref: 12737621 Clin Sci Lond < MedlineTA>Clin Sci Lond , 2003
